# Observational study on chest pain during the Covid-19 pandemic: changes and characteristics of visits to a Norwegian emergency department during the lockdown

**DOI:** 10.1186/s12873-022-00612-w

**Published:** 2022-04-02

**Authors:** Mikkel Grande, Lars Petter Bjørnsen, Lars Eide Næss-Pleym, Lars Erik Laugsand, Bjørnar Grenne

**Affiliations:** 1 Norwegian University of Science and Technology, Trondheim, Norway; 2 Clinic of Emergency Medicine and Prehospital Care, St. Olav’s University Hospital, Trondheim, Norway; 3 Department of Circulation and Medical Imaging, Norwegian University of Science and Technology, Trondheim, Norway; 4 The Norwegian Air Ambulance Foundation, Trondheim, Norway; 5 Clinic of Cardiology, St. Olav’s University Hospital, Trondheim, Norway

**Keywords:** Covid-19, Chest pain, Acute coronary syndrome, Myocardial infarction, Emergency services, Hospital, Norway

## Abstract

**Background:**

Following the spread of the Covid-19 pandemic in 2020, reports emerged on decreasing emergency department (ED) visits in many countries. Patients experiencing chest pain was no exception. The aim of the current study was to describe how the Covid-19 pandemic and the subsequential lockdown impacted the chest pain population in a Norwegian ED.

**Methods:**

All patients presenting to the ED with chest pain during the study period were included. Data were collected retrospectively from the time period January 6^th^ to August 30^th^, 2020, and compared to the corresponding period in 2019, assessing variations in the number of ED visits, severity, gender, and age.

**Results:**

Fewer patients with chest pain were seen in the ED following the national lockdown in Norway, compared to the corresponding 2019 period (week 13: 38% fewer; weeks 11–27: 16% fewer). By week 28, the rate normalized compared to 2019 levels. There was a relative increase in lower acuity patients among these patients, while fewer moderate acuity patients were seen. During the initial period following lockdown, the median age was lower compared to the corresponding 2019 period (58 years (IQR 25) vs 62 years (IQR 24), respectively). Admissions due to acute coronary syndromes (ACS) remained proportionally stable.

**Conclusions:**

Succeeding the Covid-19 outbreak and the subsequent national lockdown in Norway, fewer chest pain patients presented to the ED. Paradoxically, the patients seemed to be less severely ill and were on average younger compared to 2019 data. However, the proportion of patients admitted with ACS was stable during this period. This could imply that some patients may have failed to seek medical advice despite experiencing a myocardial infarction.

## Background

The Severe Acute Respiratory Syndrome CoronaVirus 2 (SARS-CoV-2) emerged in Wuhan, China in December 2019, and spread rapidly worldwide causing the Coronavirus Disease 2019 (Covid-19) pandemic [1]. Italy was the first country in Europe to be severely stricken by the disease, and the health care services struggled to keep up with the rapid surge of cases due to limited capacity of intensive care units [2]. Similar scenarios were expected in other European countries the following weeks.

The first case of Covid-19 in Norway was reported on February 21^st^, 2020. By March 12^th^, reported cases in Norway counted 621. In some cases, it was no longer possible to determine the source of infection. Comprehensive measures were implemented by the Norwegian Institute of Public Health (NIPH) the same day, March 12^th^, in an effort to contain the situation [3]. From April 20^th^, the preventive measures in Norway were gradually lifted as the number of new cases decreased. The incidence of Covid-19 remained low over the summer, followed by a steady increase in the number of new cases throughout August.

As Covid-19 spread across the world, a growing number of reports were published on a substantial decline in the numbers of emergency department (ED) visits [4, 5]. An initial report from the ED at St. Olav’s University Hospital presented findings consistent with this trend, depicting a general reduction in all patient groups, with no specific symptom, condition or acuity level standing out [6].

The general decline in ED visits has also been found to include patients presenting with chest pain and possible acute coronary syndrome (ACS) in several international studies [7–9]. This trend was worrisome as this patient population is prone to severe complications, such as acute myocardial infarction, heart failure, arrhythmia, and death, if there are delays in treatment. An increase in such events has been reported since the beginning of the Covid-19 pandemic [10]. Moreover, a substantial decline in the rate of hospitalization among patients with ACS has also been reported [4], and the number of cardiac arrests outside of hospitals has increased in some countries during this period [11].

The trend of a general decline in ED visits as reported internationally was confirmed in an initial report from the ED at St. Olav’s University Hospital by Bjørnsen et al. [6]. Although the report provided an overview of the situation during an early phase, it is not known how the Covid-19 pandemic and the following national lockdown affected the chest pain population specifically in this early phase and throughout the following months. Thus, the present study sought to provide insight into how the chest pain population in the ED of a Norwegian University Hospital responded to the Covid-19 pandemic and the following national lockdown.

## Materials and methods

### Clinical setting

St. Olav’s University Hospital is the local hospital in the city of Trondheim in central Norway, serving a population of approximately 300 000 inhabitants. Additionally, the hospital functions as a regional hospital in central Norway, covering more than 700 000 inhabitants. Annually, the university hospital manages more than 26 000 ED admissions in the main ED. As there is a separate ED for children, the patients managed in the main ED are primarily older than 16 years of age. The general rule is that patients must be referred to the ED by a physician, in most cases from their general practitioner (GP) or an urgent care center. The exception is situations when a patient’s condition requires emergency medical services (EMS). Therefore, the ED self-referral rate is low.

### Study design

All patients presenting to the ED at St. Olav’s University Hospital with chest pain as their chief complaint during weeks 2–35 (January 6^th^ to August 30^th^) in 2020 were included in this retrospective observational study. The most comprehensive preventive measures implemented by the government commenced from March 12^th^ (week 11) and were gradually lifted approaching the summer. As the number of ED visits in 2020 seemingly normalized compared to the 2019 data following week 27, weeks 11–27 were considered well suited to study the initial effects of Covid-19 and the preventive measures implemented by the government. The analysis is therefore focused on this period. Corresponding data from weeks 2–35 in 2019 were utilized for comparison, providing an opportunity to interpret data in light of seasonal variations and the increasing rates of patient visits to the ED over the recent years.

### RETTS triage system

All patients admitted to the ED go through an initial triage assessment, where they are assigned one out of five priority levels based on vital signs and algorithms specific for the patients’ chief complaint (emergency symptoms and signs, ESS). The Rapid Emergency Triage and Treatment System (RETTS ©, Predicare AB, Göteborg, Sweden) [12] is used both in-hospital and by the EMS. Based on this system, patients are primarily categorized as level 1 (red, highest acuity), level 2 (orange), level 3 (yellow) or level 4 (green, lowest acuity). Level 5 (blue) is used then patients present with other needs than emergency care.

### Patient inclusion

**Fig. 1:**
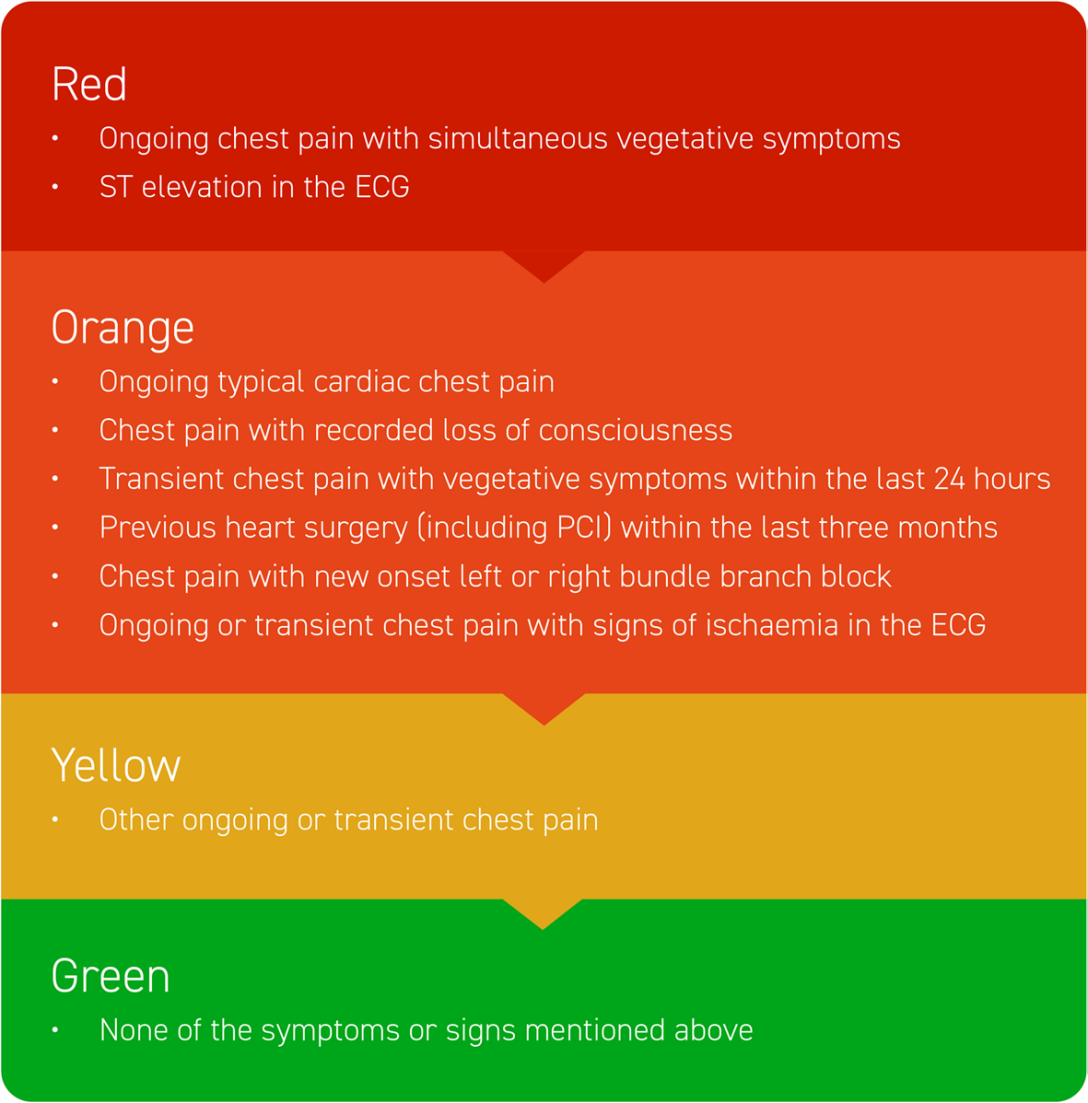
RETTS triage algorithm for chest pain, ESS #5

All patients presenting with the chief complaint of chest pain according to the RETTS ESS #5 were included in the study (Fig. 1). This approach was chosen in order to include all patients presenting with chest pain to the ED, as opposed to defining the population on the basis of discharge diagnoses.

### Data collection

Logistic data on patients referred to the ED were retrieved from Central Norway Regional Health Authority's IT (Hemit) department’s data warehouse. This included data from the local ED database (version 1.5.5. Copyright # Helse Vest IKT, Bergen, Norway). Using the administrative tool NIMES Vis (Nirvaco Medical Systems), supplementary data on the patients’ stay in hospital including discharge diagnoses and procedures were retrieved from the hospital’s patient and administration system (PAS, Hemit, 1986, version 5.2, Norway). All data were linked by an automated algorithm, anonymized, and stored on a safe hospital server.

Data on the total number of visits to the ED and the demographics of the population with regards to sex and age, was collected. The rate of patients transported to the ED by the EMS, the triage level, the in-hospital level of care, discharge diagnosis, in-hospital mortality, 30-day mortality, and the readmission rate within 30 days from the patients’ ED visit were included as surrogate markers of severity of the patents’ conditions. To account for the inconsistent reporting of how patients are transported to the ED (14.7% missing data), patients with a prehospital triage code were also included as being transported by the EMS. Regarding the remaining variables, 2.7% of patients had no recorded discharge diagnoses, 1.3% lacked data on possible readmissions and 30-day mortality, 1.0% of patients had no registered gender data and 0.3% lacked age information. These data are likely missing due to errors in the documentation process. However, the tools used for patient data collection in this study ensured a high level of accuracy to include the patients and match with their relevant clinical data. Therefore, there were generally very few missing data for the population. No data was missing for triage levels or level of care.

### ICD-10 diagnosis and patient categorization

When discharged from the ED or hospital, all patients receive one or more ICD-10 code(s) reflecting their diagnosis [13]. Each patients’ primary ICD-10 code was used to classify patients into one of four predefined groups of diagnoses within the field of cardiology: (1) Non-specific chest pain (R07.4, R07.3, and R07.2), (2) ACS (unstable angina (I20.0) and acute myocardial infarction (I21)), (3) Arrhythmia (I47, I48, I49, and R00), and (4) Other cardiac conditions categorized based on the primary discharge diagnosis, including stable angina (I20.1-I20.9. I25, I30-35, I40-43, I50-51, Q21, Q23, Z95, D15, Z03.4, Z03.5, Z45.0, and Z94.1).

### Statistics

Data were analyzed using STATA (STATA/IC 14.2, StataCorp, College Station, TX, USA). Results are reported as numbers with percentages, mean with standard deviations in normally distributed data and median with interquartile range in skewed data. The numbers of ED visits can vary substantially from day to day [6]. To reduce the impact of such periodic and random variation in the presented graphic figures, moving averages were utilized to enhance the underlying trends. Baseline 2019 data were depicted as five-week moving averages, while three-week moving averages were applied to 2020 data to capture more abrupt changes.

## Results

### Rate of ED visits due to chest pain

**Fig. 2:**
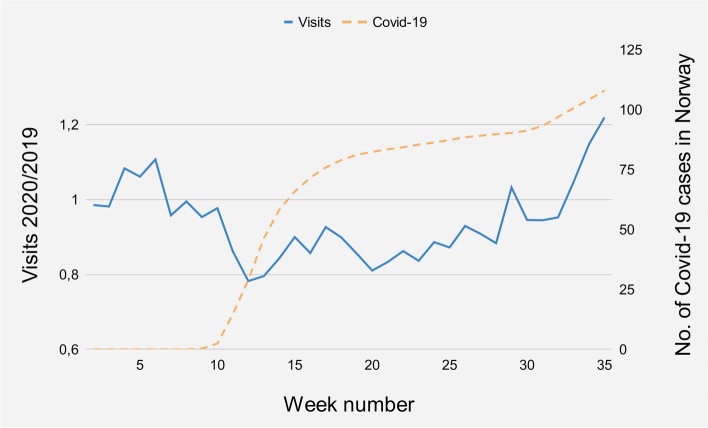
Relative trend in ED visits (2020/2019) due to chest pain throughout the study period (weeks 2–35) in relation to Covid-19 cases in Norway. Ratio for weekly ED visits in 2020 (three-week moving average) compared to 2019 (five-week moving average) is depicted with the blue solid line. The orange dashed line represents the cumulative number of Covid-19 cases in Norway during these weeks with a scale factor of 100

During the 2020 study period (January 6^th^ to August 30^th^) 1632 patients presented to the ED due to chest pain, compared to 1736 patients in the corresponding 2019 period (Table 1). In early 2020 (weeks 2–10), 498 patients presented to the ED due to chest pain, while 490 patients presented in the corresponding 2019 control period (Fig. 2). This translates to 55 (SD 8) weekly patients on average in early 2020, compared to 45 (SD 6) patients per week during the corresponding 2019 period, but higher than the 2019 average of 51 (SD 8) patients per week (weeks 2–35). From the national lockdown in week 11 to the end of the study period (weeks 11–35), there were 112 (9%) fewer patients compared to the corresponding weeks in 2019. Focusing on the period from week 11 to week 27, after which the numbers of ED visits normalized, there were 140 (16%) fewer patients compared to the same period in 2019.

**Table 1:** Overview of variations in numbers of patients presenting to the ED due to chest pain, demographic characteristics and surrogate markers of severity throughout weeks 2–35 in 2019 and 2020

	**2019**								**2020**							
	**Week 2–10**		**Week 11–27**		**Week 28–35**		**Total study period Week 2–35**		**Week 2–10**		**Week 11–27**		**Week 28–35**		**Total study period Week 2–35**	
No. of Patients	490		893		353		1736		498		753		381		1632	
No. of Patients/Week	54	(SD 6)	53	(SD 7)	44	(SD 8)	51	(SD 8)	55	(SD 8)	44	(SD 6)	48	(SD 10)	48	(SD 9)
No. of Male Patients	266	(54%)	528	(59%)	191	(54%)	985	(57%)	267	(54%)	446	(59%)	194	(51%)	907	(56%)
Median Age	61	(IQR 21)	63	(IQR 23)	64	(IQR 27)	63	(IQR 23)	60	(IQR 25)	61	(IQR 25)	61	(IQR 27)	61	(IQR 25)
< 60 years	220	(45%)	372	(42%)	146	(42%)	738	(43%)	245	(49%)	354	(47%)	177	(47%)	776	(48%)
≥ 60 years	270	(55%)	519	(58%)	205	(58%)	994	(57%)	253	(51%)	397	(53%)	199	(53%)	849	(52%)
Transportet by the EMS	188	(38%)	342	(38%)	139	(39%)	669	(39%)	154	(31%)	260	(35%)	164	(43%)	578	(35%)
Triaged Red or Orange	341	(70%)	636	(71%)	265	(75%)	1242	(72%)	313	(63%)	425	(56%)	245	(64%)	983	(60%)
Level of Care																
Outpatient Care	155	(32%)	296	(33%)	98	(28%)	549	(32%)	192	(39%)	252	(33%)	149	(39%)	593	(36%)
Admitted to Ward	293	(60%)	558	(62%)	236	(67%)	1087	(63%)	289	(58%)	474	(63%)	209	(55%)	972	(60%)
HDU/ICU	29	(6%)	32	(4%)	15	(4%)	76	(4%)	13	(3%)	18	(2%)	19	(5%)	50	(3%)
Discharge Diagnosis																
Nonspecific Chest Pain	179	(37%)	349	(39%)	136	(39%)	664	(38%)	189	(38%)	301	(40%)	159	(42%)	649	(40%)
Acute Coronary Syndrome	54	(11%)	79	(9%)	46	(13%)	179	(10%)	49	(10%)	77	(10%)	42	(11%)	168	(10%)
Arrhythmia	37	(8%)	78	(9%)	22	(6%)	137	(8%)	31	(6%)	52	(7%)	17	(4%)	100	(6%)
Other Cardiac Diagnoses	65	(13%)	137	(15%)	51	(14%)	253	(15%)	68	(14%)	108	(14%)	59	(15%)	235	(14%)
30-day mortality	10		10		2		22		7		7		3		17	
Readmission rate (1–30 days)	45	(9%)	104	(12%)	44	(12%)	193	(11%)	45	(9%)	92	(12%)	43	(11%)	180	(11%)

Concentrating on the week-to-week development, a marked decline over the three-week period from the national lockdown (weeks 11–13) was observed. Only 36 patients presented to the ED due to chest pain in week 13, thus reaching a low-point representing a 38% decline compared to 58 patients in the corresponding week in 2019. This corresponds to a 30% decline from the weekly average 2019 control period (weeks 2–35) average of 51 (SD 8), and 35% fewer patients compared to the 2020 pre-lockdown (weeks 2–10) average of 55 (SD 8).

Following the national lockdown in week 11, there were on average 11 (21%) fewer patients presenting to the ED due to chest pain per week during weeks 12–15 in 2020 compared to the same period in 2019. Compared to the pre-lockdown period in 2020 (weeks 2–10), there were on average 13 (24%) fewer weekly patients, and 9 (19%) fewer weekly patients compared to the 2019 control period (weeks 2–35). Over the next four weeks (16–19), the average number of patients per week was reduced by 8 (16%) compared to the corresponding 2019 period, and reduced by 12 patients (22%) per week compared to the early 2020 period (weeks 2–10). The marked decline in patients presenting to the ED due to chest pain during weeks 11–13 was followed by a gradual recovery until the number of patients normalized around week 28.

### Age

**Fig. 3:**
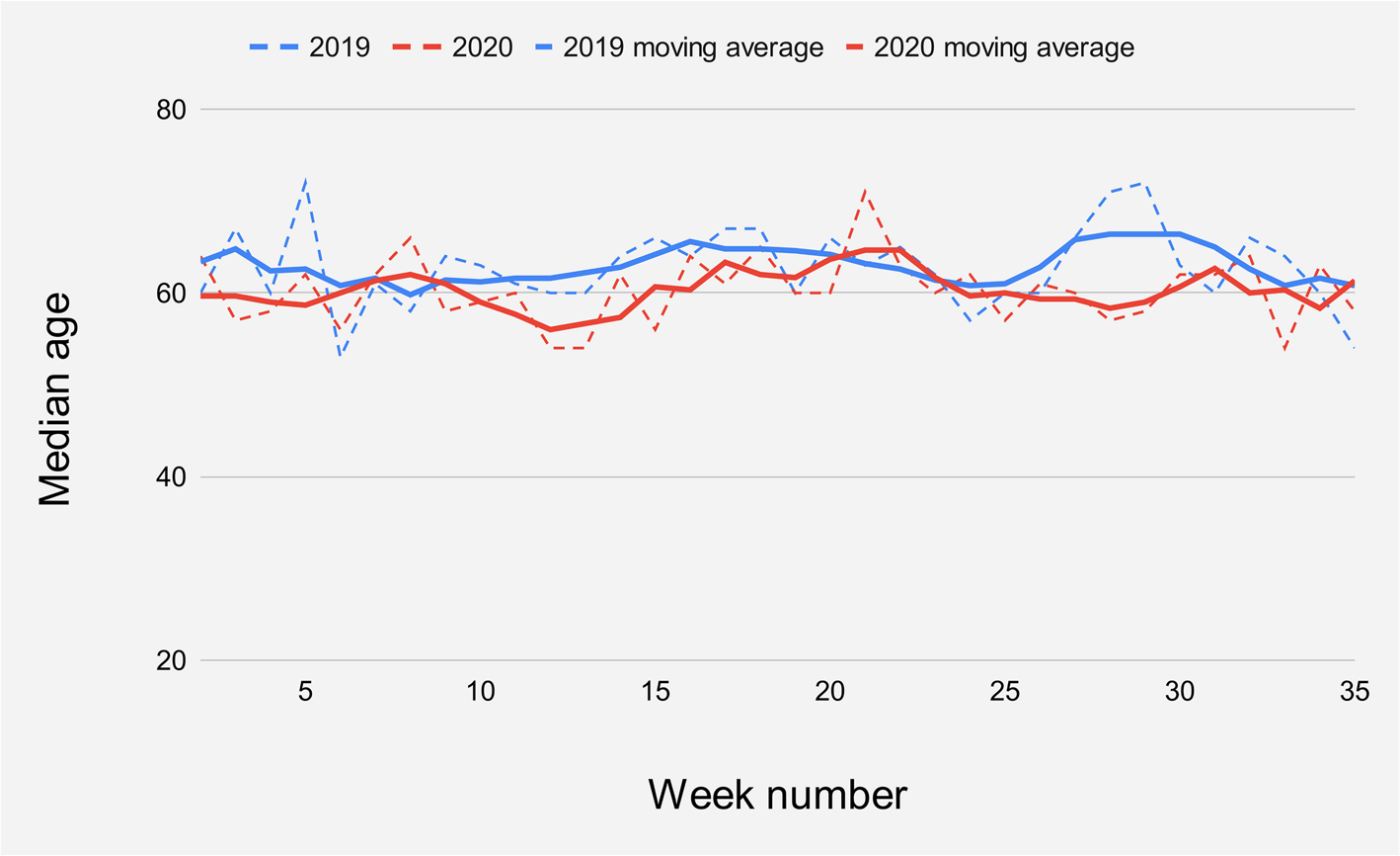
The median age of the patient population. Dashed lines reflect weekly numbers, while solid lines depict the trend as five-week moving averages for 2019 data and three-week moving averages for 2020 data respectively

The median age was 61 years (IQR 25) in 2020 compared to 63 years (IQR 23) in 2019. No apparent changes were found when comparing the Covid-19 period (weeks 11–27) with the corresponding weeks in 2019 overall, but there was an initial decline in the median age to 58 years (IQR 25) during weeks 11–15 as Covid-19 broke out in Norway (Fig. 3). The median age was 62 years (IQR 24) in the corresponding 2019 period. During the general staff holiday (weeks 28–30), the median age remained stable at 62 years (IQR 28) in 2020, but increased to 71 years (IQR 23) in 2019. There were 48 patients over 70 years old presenting to the ED in weeks 28–30 in 2020, while counting 64 patients in 2019.

### Triage level

#### RED patients

**Fig. 4:**
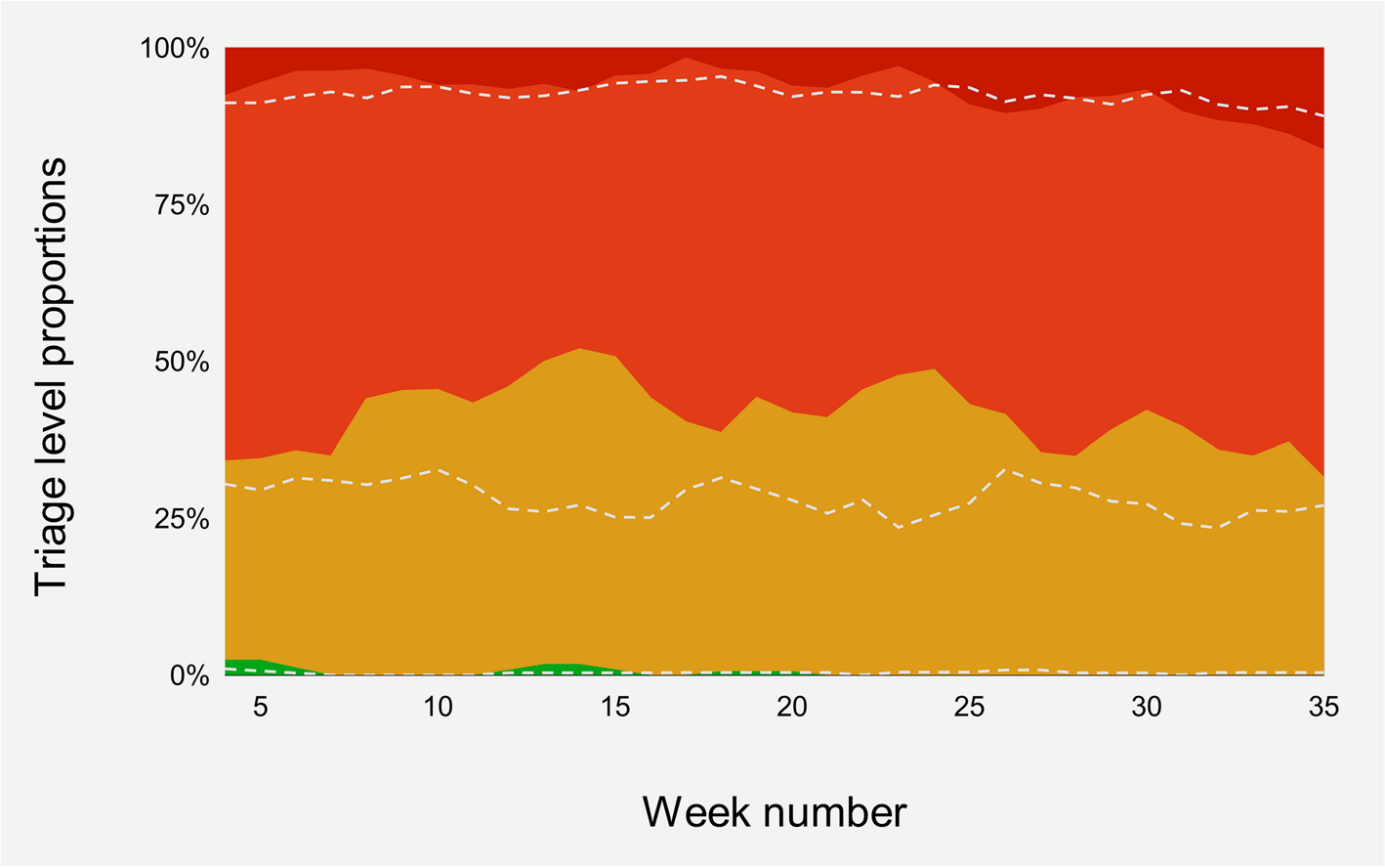
Weekly rates of patients allocated to the respective triage levels upon arrival in the ED presented as five-week moving averages for 2019 data and three-week moving averages for 2020 data respectively. Colored areas represent the 2020 data, dashed lines show corresponding 2019 levels

A total of 121 out of 1632 (7%) chest pain patients were triaged at the highest acuity level (RED) during the 2020 study period (weeks 2–35). In the 2019 control period, 133 out of 1736 (8%) were triaged at this level (Fig. 4). Focusing on weeks 11–27 in 2020, the proportion of patients at this triage level was 6% compared to 7% in the corresponding 2019 period.

#### ORANGE patients

Throughout the study period in 2020 (weeks 2–35), 862 out of 1632 (53%) patients were triaged at the second highest level (ORANGE). The corresponding numbers for 2019 were 1109 out of 1736 (64%). During weeks 11–27 in 2020, the proportion of patients at this triage level was 51% compared to 64% in the corresponding 2019 period.

#### YELLOW patients

There were 641 (39%) patients triaged at the third highest level (YELLOW) in 2020 (weeks 2–35), corresponding to 484 (28%) patients in 2019. During the Covid-19 period (weeks 11–27, 2020), 43% of the patients were triaged as YELLOW, as opposed to the 28% in the corresponding 2019 period.

#### GREEN patients

There were five patients triaged as GREEN during the 2020 study period, and ten during the corresponding 2019 period.

### Demographic, logistic and diagnostic patient characteristics

There was a majority of male patients both in 2019 (57%) and 2020 (56%). During the Covid-19 period (weeks 11–27, 2020), 260 out of 753 (35%) patients were transported to the ED by ambulance, compared to 342 out of 893 (38%) in the corresponding 2019 period. However, only 10 out of 47 (21%) patients presented to the ED during the week of the national lockdown (March 9^th^ to March 15^th^, 2020) compared to 19 out of 54 (35%) in the corresponding week in 2019.

The rate of outpatient visits increased from 549 out of 1736 (32%) in 2019 (weeks 2–35) to 593 out of 1632 (36%) in 2020 (weeks 2–35), the rate of patients admitted to hospital wards did not differ notably in the Covid-19 period (weeks 11–27, 2020) compared to the corresponding 2019 period (Table 1), and the rate of ICU admissions remained low during the study period. There were generally stable rates of patients diagnosed with nonspecific chest pain, acute coronary syndromes, arrhythmia or other cardac diagnoses during the study period. However, the rate of patients diagnosed with arrhythmias dropped from 52 out of 753 (7%) during the Covid-19 period (weeks 11–27, 2020) to 9 out of 258 (4%) in the weeks following the national lockdown in Norway (weeks 11–16).

There were 10 in-hospital deaths in the 2020 study period (week 2–35) compared to 14 in the corresponding 2019 period. The 30-day mortality was 17 in the 2020 study period compared to 22 in the 2019 control period. There was no notable change in the readmission rate.

## Discussion

**Fig. 5:**
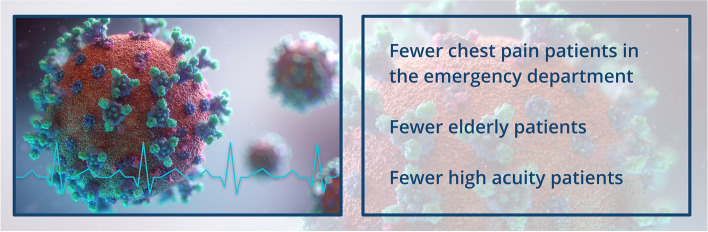
A summary of the main findings of the study

Chest pain patients presenting to the ED at a regional University Hospital in Norway were analyzed in this study, examining how this patient population responded to the Covid-19 pandemic and subsequent national lockdown. The main findings were that 1) ED visits due to chest pain decreased substantially following the outbreak of Covid-19 and subsequent national lockdown. The abrupt decline persisted almost four months before normalizing. 2) Accompanying this decline was a relative decrease in elderly patients initially, but no apparent differences among sexes were found. 3) The marked drop from ORANGE to YELLOW triage levels imply that the patients on average presented with less severe symptoms (Fig. 5).

Following the national lockdown in week 11, substantially fewer patients with chest pain presented to the ED at St. Olav’s University Hospital. The 35% decline in weekly visits following the national lockdown is compatible with the findings of a study by Myhre et al. [14]. An initial report on the ED patient population at St. Olav’s University Hospital depicted an overall decline in all ED patient groups in the same period. By week 12, there were 39% fewer ED visits compared to weeks 2–10 [6]. Thus, the decline in ED visits at St. Olav’s University Hospital seems to be slightly more pronounced in the general ED population than in chest pain patients, a trend also noted in Italian data [4]. It is however unknown whether the general ED visits continued to decline after week 12 or not, which would result in a greater difference between these groups if this was the case. Such findings were reported in Finland, where a marked drop in ED visits following the national lockdown was accompanied by stable rates of admissions due to acute myocardial infarction [15]. The other possible scenario, where the number of ED visits recover or stabilize, entail a delay in the decline of ED visits due to chest pain compared to the general ED population. A large US multicenter study reported a faster recovery of visits due to serious cardiac conditions compared to overall ED visits [16]. Similarly, acute myocardial infarction cases recovered relatively faster also in Germany [5]. This could imply that the impact of Covid-19 has been greater in other patient populations compared to the chest pain population.

The number of ED visits due to chest pain at St. Olav’s University Hospital gradually recovered from the initial decline, normalizing in week 28 compared to 2019 data. There were 16% fewer patients presenting to the ED due to chest pain throughout weeks 11–27 compared to 2019, corresponding well to the overall reduction in ACS admissions seen in England in the same period [17]. The decline was however greater at its peak in England than in the current study [7], as was also seen in other European studies (39 to 45%) [4, 8]. Focusing on the initial four-week period (week 12 to 15) following the national lockdown in Norway, a 19% reduction was found. A German multicenter study reports a 39% reduction in admissions due to acute myocardial infarction during the same weeks [18], in line with Mafham et al.’s findings in England [7]. Although the study populations are not directly comparable, this suggests a greater and longer lasting initial decline in ACS admissions in Germany and England. Despite the decline being evident in England from week 10 [7], one week earlier than in the ED at St. Olav’s University Hospital [6], the national lockdown in Norway was implemented 11 days earlier than in England. One might speculate that the early initiation of a national lockdown in Norway contributed to a less severe course of Covid-19 throughout the study period. This could have resulted in fewer deaths from cardiovascular disease and lower total mortality in Norway during the lockdown period as reported by the NIPH, contrary to the development in many European countries [19]. According to Mafham et al., weekly ACS admissions had approximately recovered by August [17], a month later than in our data. In both Italy and Germany, the admissions and prevalence of acute myocardial infarction normalized in May [5, 20]. Considering that the delayed normalization in England also is seen for all acute myocardial infarction admissions (not only in the total ACS population) [17], it is likely that there is a genuine difference in the timing of normalization.

Relatively fewer elderly patients (over 60 years) presented to the ED at St. Olav’s University Hospital due to chest pain following the national lockdown in Norway (weeks 11–15). While similar findings were reported in a general ED population in the US [21], the opposite was found in New Zealand [22]. Considering the association between increasing age and increasing mortality from Covid-19 [23], one could expect the elderly to be more afraid of the consequences of acquiring Covid-19. Furthermore, the fear of Covid-19 infection is regarded as one of the most important factors causing the decline in ED visits [6, 8, 11]. This could potentially explain the disproportionate decline in elderly patients in our data. Additionally, emergency care of elderly patients is often initiated by their relatives [5]. The social distancing measures implemented to contain the spread of SARS-CoV-2 presumably resulted in elderly patients being isolated from their relatives, thus unintentionally preventing ED referrals of many elderly patients [5, 11]. Counteracting measures should therefore be considered to ensure adequate acute health care services for the elderly in similar situations in the future.

It is possible that the same mechanisms were at play during the general staff holiday (weeks 28–30), where relatively fewer elderly patients visited the ED due to chest pain compared to 2019. While the median age remained stable at 60 throughout weeks 28–30 in 2020, it peaked at 72 in week 29 in the corresponding 2019 period. Further examinations of this unexpected finding by differentiating the study population into 10-year age groups, revealed a substantial rise in the number of patients over 70 years old. A similar rise did not take place in 2020, where only 48 patients over 70 years presented to the ED in this period compared to 64 in 2019. There were profound declines in the total ED visits due to chest pain throughout this period both in 2019 and 2020, thus it seems as though the surge of elderly patients in 2019 were compensated by an increase in younger patients in 2020. This increase could be caused by more younger people staying at home due to travelling restrictions, contributing to an impression of normalizing numbers of ED visits during the general staff holiday.

Generally, chest pain patients sought out medical advice equally throughout the study period regardless of severity. The same trend was demonstrated in the general ED population at St. Olav’s University Hospital early on (6). Compatible findings are reported in the US (11, 23), although Italian data suggest that the patients on average were more severely ill (4). This could be attributed to the immense pressure on the Italian healthcare system in an early phase of the pandemic (24). In the current study, the proportion of patients triaged as ORANGE dropped (64% to 51%) during the Covid-19 period (weeks 11–27, 2020) compared to 2019, while patients triaged as YELLOW increased (28% to 43%). This implies that chest pain patients presenting to the ED at St. Olav’s University Hospital during the Covid-19 period were less severely ill than the previous year. The opposite was noted in New Zealand regarding triage levels, where the proportion of low acuity presentations decreased significantly (21). Considering that other parameters indicating severity were more in line with the current study, this is difficult to interpret. One might speculate how well the triage levels reflect the severity of these cases. The Norwegian Directorate of Health reported an 18% increase in patient contacts with GPs, and a 34% increase in the use of urgent care centers nationally during March 2020 compared to March 2019 [24]. In line with the gatekeeping role of the GP, one might speculate whether the threshold for referring patients to the ED increased in this period of high demand. Presumably, this would have resulted in relatively fewer low acuity presentations, which is not found in our data. This suggests that other mechanisms were more prominent in causing the decline in patients presenting to the ED. Moreover, no in-hospital mortality was recorded in the study population for several months during the Covid-19 period. This result could be coincidental considering the small data size, but it is in line with reports of normal or lower excess mortality rates in Norway in this period [25]. Other surrogate markers of severity used in this study, such as discharge diagnosis and level of care, did not indicate changes in the degree of severity, thus making the interpretation of these findings difficult.

### Limitations

Challenges regarding completeness of data and control over procedures for data collection are prominent when doing a retrospective register study. Additionally, such studies are not suited for concluding on causality. Thus, it cannot be concluded whether the decline in ED visits is due to a true decline in the incidence of chest pain or not based on this study. As the aim of the study was to provide insight into how the chest pain population in a Norwegian ED responded to the national lockdown and reopening in Norway following the spread of the Covid-19 pandemic, data on the subsequent outbreaks during the fall have not been gathered or analyzed. Including data from more prior years would provide an opportunity to account for seasonal variations and the increasing numbers of ED visits. The interpretation of more subtle changes in the study population could also become more conclusive. The inclusion process, where patients assessed after the RETTS [12] triage algorithm for chest pain were included, has some weaknesses. Patients with atypical presentations of ACS, such as dyspnea, palpitations or abdominal pain, may have been excluded if assessed after other triage algorithms. Although less likely, it is possible that hemodynamically affected patients presenting with chest pain were triaged based on vital parameters if suggesting a higher level of triage than the algorithm for chest pain. The current study does not differentiate between ST-Elevation Myocardial Infarction (STEMI) and Non-ST-Elevation Myocardial Infarction (NSTEMI). Although equally decreased rates of NSTEMI and STEMI have been reported [26], international studies imply a more profound decline in NSTEMI [7–9]. Furthermore, Pines et al. reported no clear evidence for a decline in STEMI in this period [16]. In the ED at St. Olav’s University Hospital, STEMI patients often bypass the ED through accelerated pathways. It is thus possible that an increase in patients with STEMI could go undetected in this study.

## Conclusion

The number of chest pain patients presenting to the ED at St. Olav’s University Hospital declined considerably following the outbreak of Covid-19 and subsequent national lockdown. This decline was however not as pronounced as in many other European countries. The decline impacted the whole study population but seems to have affected the elderly more than the general population in the early phase. This suggests a need for increased preparedness targeting the elderly in similar situations in the future. Moreover, the shift toward lower triage levels suggests that the patients on average were less severely ill. Further studies will be needed to assess if the observed changes had any influence on morbidity and mortality.

## Data Availability

The dataset used and analyzed during the current study are available from the corresponding author on reasonable request.

## Abbreviations

ACS: Acute Coronary Syndrome
CI: Confidence Interval
Covid-19: Coronavirus Disease 2019
ED: Emergency Department
EMS: Emergency Medical Services
ESC: European Society of Cardiology
ESS: Emergency Symptoms and Signs
GP: General Practitioner
Hemit: Central Norway Regional Health Authority's IT department
ICU: Intensive Care Unit
IQR: Interquartile Range
NIPH: National Institute of Public Health
NSTEMI: Non-ST-Elevation Myocardial Infarction
PCI: Percutaneous Coronary Intervention
REK: Regional Health Research Ethics Committee
RETTS: Rapid Emergency Triage and Treatment System
SARS-CoV-2: Severe Acute Respiratory Syndrome Corona-Virus 2
SD: Standard Deviation
STEMI: ST-Elevation Myocardial Infarction
